# Precision Enhancement of Multi-Source Integrated Navigation via Solar Disk Differential Velocity Compensation

**DOI:** 10.3390/s26134273

**Published:** 2026-07-05

**Authors:** Yueqing Huang, Xiaolin Ning

**Affiliations:** School of Instrumentation Science & Opto-Electronics Engineering, Beihang University, Beijing 100191, China; celenavbuaa@buaa.edu.cn

**Keywords:** doppler velocity sensor, multi-sensor fusion, solar differential rotation, unmodeled system bias, integrated navigation

## Abstract

**Highlights:**

**What are the main findings?**

- A solar disk differential velocity (SDV) method is proposed to eliminate the deterministic system bias (up to 2.07 km/s) caused by the assumption of a uniform radial velocity distribution across the solar disk.
- A multi-source fusion framework is constructed by integrating Doppler measurements from the solar disk and dual stars with SINS to enhance system observability.
- Simulation results demonstrate that the proposed method significantly improves navigation accuracy, reducing position errors from the kilometer level to the range of tens to hundreds of meters.

**What are the implications of the main findings?**

- Modeling the solar disk’s differential rotation effectively compensates for system bias, providing a high-precision solution for autonomous navigation of vehicles.
- The SDV-DVS/SINS integrated system exhibits high robustness against external disturbances, ensuring stable and precise navigation for high-dynamic vehicles.

**Abstract:**

The strapdown inertial navigation system (SINS) suffers from cumulative error growth, where velocity and acceleration drifts cause position errors to grow quadratically. While multi-sensor fusion using Doppler Velocity Sensors (DVS) can correct these drifts, traditional methods often assume a uniform radial velocity across the solar disk, thereby ignoring the differential surface velocities caused by solar rotation. This mismatch introduces a significant unmodeled system bias with a mean value of approximately 1.45 km/s and an upper bound of up to 2.07 km/s, which is orders of magnitude larger than the standard measurement error (~1 m/s). Such a dominant deterministic error leads to severe filter divergence, undermining the reliability of the entire integrated navigation system. This mismatch introduces unmodeled system bias, degrading filter performance. To address this issue, this paper proposes a solar disk differential velocity method within a multi-source fusion framework. By constructing a velocity measurement model that reflects the Sun’s actual geometric and rotational characteristics, this method effectively eliminates the deterministic system bias. Combined with dual-star Doppler measurements, the method enhances the observability of 3D velocity errors. Simulation results show that the proposed method significantly improves navigation accuracy compared to the traditional method. Position errors are reduced from kilometer-level magnitudes to the range of tens to hundreds of meters, with velocity errors remaining within 10^−2^–10^−1^ m/s.

## 1. Introduction

With the continuous development of the aerospace industry, aerospace vehicle navigation technology increasingly plays a pivotal role in military defense, economic development, and information and communication sectors, serving as an indispensable foundation for national strategies and scientific and technological advancement [1]. To minimize the dependence of navigation data on ground-based facilities and improve the feasibility and autonomy of vehicles during high-dynamic flights, it is particularly important to develop autonomous navigation technology for vehicles [2,3]. The strap-down inertial navigation system (SINS) is widely used in aerospace autonomous navigation due to its strong autonomy, high update rate, and independence from external signals [4,5]. However, due to the integral calculation used by SINS to obtain navigation information of the vehicle, error accumulation is inevitable during the entire flight duration of SINS, resulting in drift of navigation error over time [6,7]. Especially, velocity drift leads to position error growing approximately quadratically, seriously affecting navigation accuracy and stability [8]. Therefore, introducing highly reliable external velocity information for error compensation of SINS is crucial for improving navigation accuracy and system stability. Traditional methods usually rely on velocity measurements from stars, Global Navigation Satellite System (GNSS), or radar to correct the velocity errors of SINS [9,10,11]. However, these artificial signal sources are often susceptible to interference and heavily dependent on communication links, making them unsuitable for high-autonomy, all-weather navigation applications [12,13]. As the closest natural radiative source to Earth, the Sun offers high-intensity signals and all-weather observability. Without relying on external infrastructure, it can provide continuous, stable, and reliable observational data, and has thus attracted increasing attention in the field of autonomous navigation in recent years [14,15]. In recent years, several studies have introduced the Sun into Doppler velocity measurement systems to aid inertial navigation systems in correcting velocity drift, improving navigation system accuracy and stability [16,17,18].

Studies show that the velocity across the solar disk varies significantly with latitude, which is due to the Sun’s differential rotation [19,20]. Although the extended source characteristic of the Sun has been recognized, traditional methods often assume a uniform radial velocity across the solar disk, thereby neglecting the different velocity distributions generated by solar differential rotation. Consequently, these methods fail to accurately capture the velocity variations at different latitudes, introducing a systematic bias into the measurement model. This bias appears as a slowly varying error with a nonzero mean, reaching the order of kilometers per second. It can readily cause filter divergence during integrated navigation, thereby significantly degrading the accuracy and robustness of the navigation system.

This study proposes a high-precision multi-source integrated navigation framework that incorporates a solar disk differential velocity to address unmodeled system biases (SDV-DVS/SINS). In this method, the SINS error equation serves as the state model, and Doppler velocity from the point on the solar disk and two stars are used as measurements to provide high-precision velocity information. The DVS measurement model, derived in detail, reflects the solar rotation and accurately describes the differential velocities at different latitudes. A Doppler velocity measurement model for stars is also derived. Then, to demonstrate the effectiveness of the newly proposed method, the performances of the traditional DVS/SINS and the newly proposed method were compared, and the influence of the measurement error and different stars observed in the navigation were evaluated by simulations.

After the introduction, Section 2 provides an analysis of the unmodeled system bias. Section 3 presents the state and measurement models of the proposed SDV-DVS/SINS integrated navigation. Section 4 analyzes the performance of the proposed method through simulations. Section 5 presents the discussion, and Section 6 concludes the paper.

## 2. Unmodeled System Bias Under the Uniform Radial Velocity Distribution Across the Solar Disk Assumption

### 2.1. Analysis of Unmodeled System Bias

In the traditional DVS/SINS integrated navigation method, the radial velocity distribution across the solar disk is assumed to be uniform. However, studies have shown that the Sun exhibits differential rotation, with its surface angular velocity depending on heliographic latitude [21], as expressed by the equations below.

(1) $$w\left(\varphi \right)=w_{0}+w_{1}\sin^{2}\left(\varphi \right)+w_{2}\sin^{4}\left(\varphi \right)$$

where φ is the heliographic latitude, $w_{0}=14.713^{\circ }/d$, $w_{1}=-2.396^{\circ }/d$, $w_{2}=-1.787^{\circ }/d$. As a result, the linear velocity on the solar surface exhibits large variations: approximately 2.0 km/s near the equator and less than 0.5 km/s in polar regions, yielding a maximum velocity gradient of 1.5 km/s across the disk.

This pronounced latitude-dependent velocity variation is fundamentally inconsistent with the uniform-velocity assumption of the traditional method. Consequently, the traditional DVS measurement model inevitably introduces an unmodeled system bias, which can be expressed as

(2) $$\Delta v_{sys}={\bar{v}}_{disk}-v_{center}$$

where $v_{center}$ is the centroid velocity and ${\bar{v}}_{disk}$ represents the weighted average of the linear velocity across the observed region. This can be expressed as

(3) $${\bar{v}}_{disk}=\frac{\int_{\Omega }v_{LOS}\left(r\right)\cdot W\left(r\right)d\Omega }{\int_{\Omega }W\left(r\right)d\Omega }$$

where Ω represents the field of view (FOV) of the spectrometer, $W\left(r\right)$ the weighting function, and $v_{LOS}\left(r\right)$ is the projection of the velocity vector onto the line of sight.

**Figure 1:**
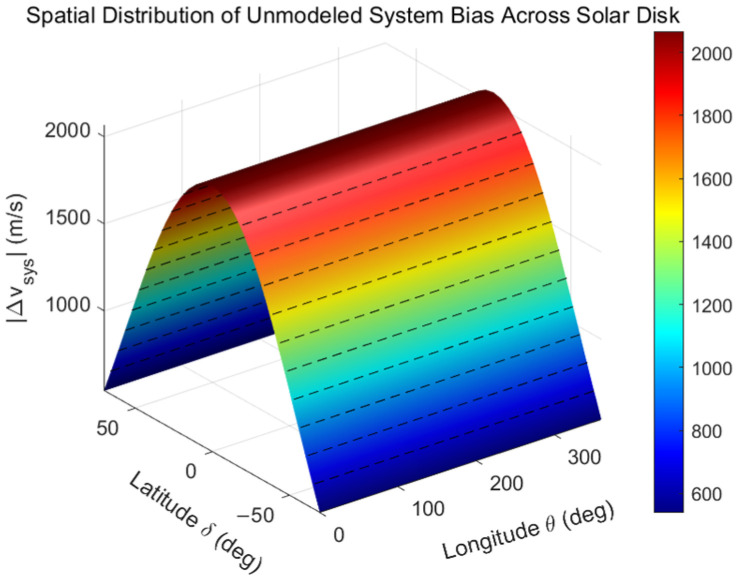
Spatial distribution of the systematic velocity error with the latitude–longitude axis across the solar disk.

As shown in Figure 1, the system bias distribution over the solar disk is obtained by traversing latitudes from −70° to 70° and longitudes from 0° to 360°. The resulting bias field exhibits a clear latitude-dependent pattern, which is directly induced by the actual differential rotation of the Sun. The velocity bias is most pronounced between equatorial and high-latitude regions.

This comprehensive quantification of the unmodeled system bias exposes the fundamental limitation of the traditional DVS/SINS method and highlights the necessity of the proposed SDV-DVS/SINS approach. By incorporating the actual rotational characteristics of the solar surface into the measurement model, the proposed method eliminates this critical modeling deficiency and enhances the accuracy and robustness of navigation.

### 2.2. Innovation Analysis for Unmodeled System Bias

In integrated navigation filtering, innovation is used to measure the deviation between actual measurement and model-predicted measurement. For a correctly modeled measurement model, the innovation should exhibit zero mean, white noise characteristics, and statistical independence, which are fundamental conditions for the optimality of the Kalman filter. However, in the traditional point source model, the solar differential rotation is not modeled, and the actual innovation becomes the following:

(4) $$z_{\mathrm{trad}}=l^{T}\left(v_{disk}-v_{center}\right)+v_{m}=l^{T}\Delta v_{sys}+v_{m}$$

where l is the direction vector, $v_{m}$ denotes the measurement error, and $\Delta v_{sys}$ is the deterministic model bias introduced by the assumption of a uniform radial velocity distribution across the solar disk.

Accordingly, the mathematical expectation of the innovation is $E\left[z_{trad}\right]=l^{T}\Delta v_{sys}$, indicating that the innovation no longer maintains a zero mean characteristic. These biased innovations mislead the filter, causing it to interpret the systematic model error as actual state error. As a consequence, the velocity states are continuously miscorrected, and the induced error propagates through integration to the position states, leading to a quadratic growth of position error over time. Such accumulated bias may degrade the stability of the filter and even cause divergence. In Section 4, the innovation performance of the traditional method and the proposed SDV-DVS/SINS method will be further compared through simulations.

## 3. System Model of the Proposed SDV–DVS/SINS Integrated Navigation

In this section, the state model and the measurement model of the proposed method are introduced. The coordinate systems used in this paper are defined as follows. The geocentric inertial coordinate system (i-frame) is an Earth-centered inertial frame. The heliocentric inertial coordinate system (HCI-frame) is a Sun-centered inertial frame. The e-frame is defined by the Earth-centered Earth-fixed (ECEF) coordinates. This study uses the geographic coordinate system (E–N–U coordinate system) as the n-frame. The body coordinate system (b-frame) is fixed to the vehicle and moves with it.

### 3.1. State Model

The error equation of SINS is adopted as the system state model, which can be expressed by Equation (5):

(5) $$\left\{\begin{array}{l} \dot{\phi }=\phi \times \omega_{in}^{n}+\delta \omega_{in}^{n}-C_{b}^{n}\delta \omega_{ib}^{b}\\ \delta {\dot{v}}^{n}=-\phi \times f^{n}+v^{n}\times \left(2\delta \omega_{ie}^{n}+\delta \omega_{en}^{n}\right)-\left(2\omega_{ie}^{n}+\omega_{en}^{n}\right)\times \delta v^{n}-C_{b}^{n}\nabla \\ \delta {\dot{r}}^{n}=M_{1}\delta v^{n}+M_{2}\delta r^{n}\\ \dot{\varepsilon }=0\\ \dot{\nabla }=0 \end{array}\right.$$

where $\phi ={\left[\phi_{E},\phi_{N},\phi_{U}\right]}^{T}$ is the misalignment angle of the SINS. $\omega_{in}^{n}$ is the rotational angular velocity vector of the n-frame relative to the i-frame in n-frame, and $\omega_{in}^{n}=\omega_{ie}^{n}+\omega_{en}^{n}$. $\omega_{ie}^{n}$ and $\omega_{en}^{n}$ are the rotational angular velocity vectors of e-frame to the i-frame in the n-frame and those of n-frame to the e-frame in the n-frame, respectively. $\delta \omega_{in}^{n}$ and $\delta \omega_{ib}^{b}$ represent the biases of $\omega_{in}^{n}$ and $\omega_{ib}^{b}$. $\delta \omega_{ie}^{n}$ and $\delta \omega_{en}^{n}$ represent the biases of $\omega_{ie}^{n}$ and $\omega_{en}^{n}$. $C_{b}^{n}$ is the transformation matrix of the b-frame into the n-frame. $f^{n}={\left[f_{x}^{n},f_{y}^{n},f_{z}^{n}\right]}^{T}$ is the accelerometer output in frame n. $v^{n}={\left[v_{E},v_{N},v_{U}\right]}^{T}$ is the velocity in the eastward, northward, and upward directions, and $\delta v^{n}={\left[\delta v_{E},\delta v_{N},\delta v_{U}\right]}^{T}$ is the velocity error of $v^{n}$. $\delta r_{n}={\left[\delta L,\delta \lambda ,\delta h\right]}^{T}$ is the position error in the latitude, longitude, and height, respectively. $\varepsilon ={\left[\varepsilon_{x},\varepsilon_{y},\varepsilon_{z}\right]}^{T}$ is the gyro drift. $\nabla ={\left[\nabla_{x},\nabla_{y},\nabla_{z}\right]}^{T}$ represents the accelerometer bias.

$$M_{1}=\left[\begin{array}{ccc} 0 & \frac{1}{R_{M}+h} & 0\\ \frac{\sec L}{R_{N}+h} & 0 & 0\\ 0 & 0 & 1 \end{array}\right]\;M_{2}=\left[\begin{array}{ccc} 0 & 0 & \frac{v_{N}}{{\left(R_{M}+h\right)}^{2}}\\ \frac{v_{E}}{\left(R_{N}+h\right)\sec L\tan L} & 0 & \frac{v_{E}}{{\left(R_{N}+h\right)}^{2}\sec L}\\ 0 & 0 & 0 \end{array}\right]$$

where L,h is the longitude and height of the vehicle, respectively. $R_{M}=R_{e}\left(1-2e+3e\sin^{2}L\right)$, and $R_{N}=R_{e}\left(1+e\sin^{2}L\right)$. $R_{e}$ is the radius of the Earth and e is the eccentricity.

Assuming that the state vector is $X={\left[\phi_{E},\phi_{N},\phi_{U},\delta v_{E},\delta v_{N},\delta v_{U},\delta L,\delta \lambda ,\delta H,\varepsilon_{E},\varepsilon_{N},\varepsilon_{U},\nabla_{E},\nabla_{N},\nabla_{U}\right]}^{T}$, the state model using the error equation can be expressed as follows:

(6) $$\dot{X}\left(t\right)=F\left(t\right)X\left(t\right)+W\left(t\right)$$

where $F\left(t\right)$ is the state transformation matrix and $W\left(t\right)$ is the process noise.

### 3.2. Measurement Acquisition

To obtain the Doppler velocity measurement of the point on the solar disk, a spectrometer is used to measure the Doppler shift in the solar spectrum. The relationship between the radial velocity and the Doppler shift can be expressed as follows:

(7) $$v_{r}=c\frac{f_{r}-f_{e}}{f_{e}}+v_{m}$$

where c is the speed of light, $f_{r}$ and $f_{e}$ are the received and emitted spectral frequency, respectively, and $v_{m}$ denotes the measurement error of the spectrometer.

In this study, the spectrometer is installed on the side of a quadripyramid. The installation angle δ of the spectrometer determines the projection position of the Line-of-Sight (LOS) on the solar disk. Let R be the radius of the circumcircle on the bottom of the quadripyramid and h be the height of the quadripyramid, then

(8) $$\delta =\arctan\left(\frac{R}{h}\right)$$

### 3.3. Measurement Model

#### 3.3.1. Doppler Velocity Measurement Model of the Solar Disk Point

The Doppler velocity measurement model of the point on the solar disk can be written as follows:

(9) $$v_{rq}={\left(v_{pq}^{i}\right)}^{T}\cdot l_{ps}^{i}+v_{m}$$

where $v_{pq}^{i}$ is the velocity of the vehicle relative to the solar observation point in the i-frame, $l_{ps}^{i}$ the direction vector of the vehicle relative to the Sun in the i-frame, and $v_{m}$ is the measurement error of the spectrometer.

According to the relative motion relationship, Equation (9) can be expressed as follows:

(10) $$v_{rq}={\left(v_{es}^{i}+v_{sq}^{i}-v_{ep}^{i}\right)}^{T}\cdot l_{ps}^{i}+v_{m}$$

where $v_{es}^{i}$ is the velocity of the Sun in the i-frame, $v_{sq}^{i}$ is the surface linear velocity of the solar disk point caused by solar rotation, and $v_{ep}^{i}$ is the vehicle velocity in the i-frame.

The surface linear velocity due to solar rotation is written as

(11) $$v_{sq}^{i}=C_{HCI}^{i}\cdot \left(w\times r_{sq}^{HCI}\right)$$

where $w={\left[\begin{array}{ccc} 0 & 0 & w\left(\varphi \right) \end{array}\right]}^{T}$ is the solar rotation angular velocity determined by the solar differential rotation model, φ is the solar latitude of the observation point, $r_{sq}^{HCI}$ is the position vector of the observation point relative to the solar center in the HCI-frame.

The spacecraft velocity in the inertial frame is obtained as

(12) $$v_{ep}^{i}=C_{n}^{i}\cdot v^{n}$$

where $C_{n}^{i}$ is the transformation matrix of the n-frame to i-frame, $v^{n}=v_{\mathrm{SINS}}-\delta v^{n}$ is the corrected spacecraft velocity, with $v_{\mathrm{SINS}}$ the SINS velocity output and $\delta v^{n}$ the estimated velocity error.

Thus, the measurement equation can be written as

(13) $$v_{rq}={\left(v_{es}^{i}+v_{sq}^{i}-C_{n}^{i}\cdot \left(v_{\mathrm{SINS}}-\delta v^{n}\right)\right)}^{T}\cdot l_{ps}^{i}+v_{m}$$

Using the Doppler velocity of the solar disk point as the measurement, denoted by $Z_{1}=v_{rq}$, we get

(14) $$Z_{1}\left(t\right)=v_{rq}\left(t\right)=h_{1}\left(X\left(t\right)\right)+v_{1}\left(t\right)$$

where $h_{1}\left(\cdot \right)$ denotes the SDVC-DVS measurement function and $v_{1}\left(t\right)$ is the measurement error.

#### 3.3.2. Doppler Velocity Measurement Model of Dual Stars

For two stars (Star-1 and Star-2), the Doppler velocity measurement models are expressed as

(15) $$v_{rs-j}={\left(v_{ps-j}^{i}\right)}^{T}\cdot l_{ps-j}^{i}+v_{m}$$

where $v_{ps-j}^{i}$ is the velocity of the vehicle relative to Star−j in the i-frame, $l_{ps-j}^{i}$ is the LOS unit vector to Star−j.

The relative velocity can be written as

(16) $$v_{ps-j}^{i}=v_{es-j}^{i}-v_{ep}^{i}$$

where $v_{es-j}^{i}$ is the i-frame velocity of Star−j, $v_{ep}^{i}$ is the velocity of the vehicle in the i-frame, which can be obtained from Equation (12).

Thus, the Doppler velocity measurement becomes

(17) $$v_{rs-j}={\left(v_{es-j}^{i}-C_{n}^{i}\cdot \left(v_{\mathrm{SINS}}-\delta v^{n}\right)\right)}^{T}\cdot l_{ps-j}^{i}+v_{m}$$

Adopting the Doppler velocities of two stars as the measurements, which can be expressed as $Z_{2}={\left[v_{rs-1},v_{rs-2}\right]}^{T}$. Then, the measurement model can be expressed as follows:

(18) $$Z_{2}\left(t\right)=v_{rs-j}\left(t\right)=h_{2}\left(X\left(t\right)\right)+v_{2}\left(t\right)$$

where $h_{2}\left(\cdot \right)$ denotes the dual-star Doppler measurement function and $v_{2}\left(t\right)$ represents the corresponding measurement error.

#### 3.3.3. Unified Measurement Model of the SDV-DVS/SINS Integrated Navigation

Let the measurement vector and measurement error of the SDV-DVS/SINS integrated navigation system be $Z_{3}={\left[Z_{1},Z_{2}\right]}^{T}$ $v_{3}={\left[v_{1},v_{2}\right]}^{T}$. Accordingly, the measurement equation of the proposed SDV-DVS/SINS integrated navigation system can be expressed as

(19) $$Z_{3}\left(t\right)=h_{3}\left(X\left(t\right)\right)+v_{3}\left(t\right)$$

where $h_{3}\left(\cdot \right)$ is the measurement function combining both the solar disk point Doppler velocity and the dual-star Doppler measurements.

From a geometric perspective, a single Doppler source (the Sun) only measures a 1D velocity projection along its line-of-sight (LOS), leaving the perpendicular components unobservable. Introducing two additional navigation stars expands the observation baseline to three distinct LOS vectors. When these three multi-source vectors are non-coplanar, the combined measurement matrix fulfills the full-rank condition, securing complete 3D velocity observability. Furthermore, this geometry directly dictates the estimation accuracy: as the intersection angles between the three LOS vectors approach 90° (mutually orthogonal), which maximizes the observability and navigation accuracy.

**Figure 2:**
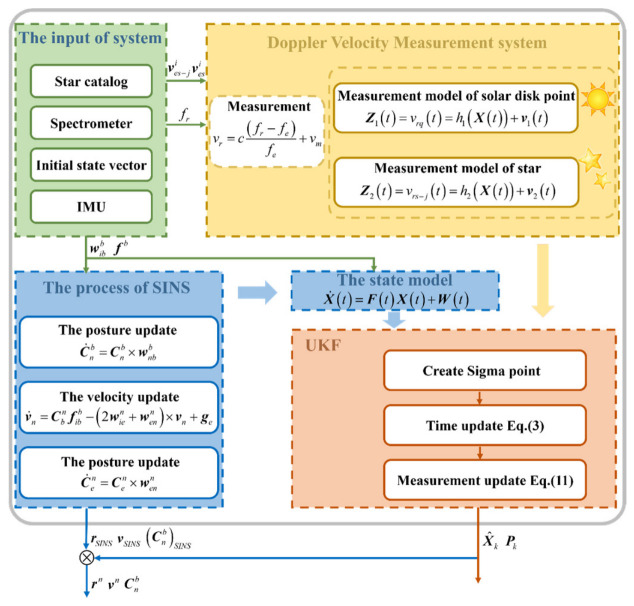
Scheme of the SDV-DVS/SINS integrated navigation.

Figure 2 presents the overall architecture of the proposed SDV-DVS/SINS integrated navigation system. As illustrated by the data flow, the system inputs are processed via the SINS mechanization loop to formulate the state model (Equation (5)). Concurrently, based on the geometric observation relationships, the multi-source measurement models are established to construct the solar disk point measurement model $Z_{1}\left(t\right)$ and the star Doppler measurement model $Z_{2}\left(t\right)$. Finally, both the state model and the combined measurements are fed into the UKF estimation loop for time update and measurement update, outputting the corrected, high-precision navigation solutions.

## 4. Simulation and Analysis

### 4.1. Simulation Conditions

**Figure 3:**
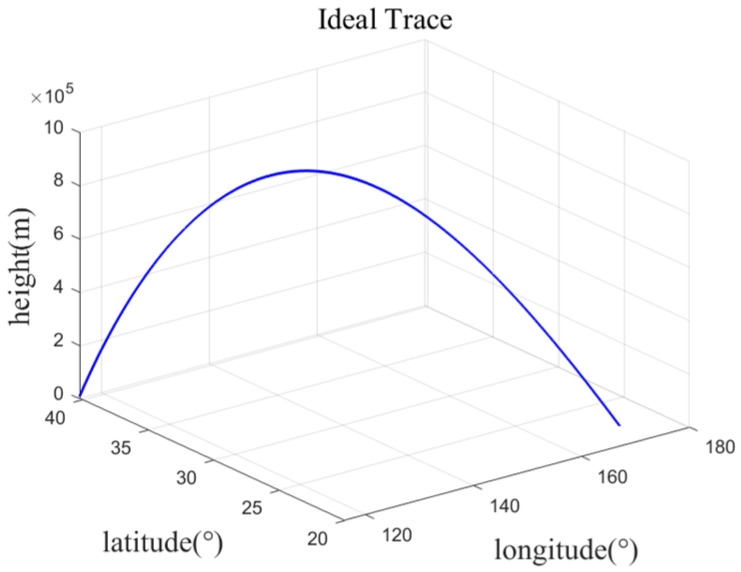
Ideal trace of the vehicle.

The ideal trajectory of the vehicle was obtained using the Satellite Tool Kit (STK). Figure 3 shows the ideal trace of the vehicle obtained by STK and the parameters of the traces are shown in Table 1. The parameters of the stars are listed in Table 2. The initial position error, velocity error, and platform misalignment angle were set to ${\left[50,50,50\;m\right]}^{T}$, ${\left[0,0,0\;m/s\right]}^{T}$, and ${\left[20^{\prime \prime },20^{\prime \prime },20^{\prime \prime }\right]}^{T}$, respectively. The gyroscope drift was 0.01 °/h, and the random noise was 0.001 ${}^{\circ}/\sqrt{h}$. The accelerometer bias was set to 100 μg, and the random noise was 100 $\mu g/\sqrt{\mathrm{HZ}}$. According to [22], the operational DVS measurement accuracy can reach 1.93 m/s. Thus, in this study, the measurement error of the DVS was set to 2 m/s. Table 3 shows the corresponding filter parameters. 

### 4.2. Innovation Analysis

To evaluate the impact of the unmodeled system bias introduced by the traditional uniform radial velocity distribution across the solar disk assumption on filter performance, this section compares the innovations of the traditional DVS/SINS method with those of the proposed SDV-DVS/SINS method. In theory, when the measurement model is correctly specified and free of systematic errors, the innovations are expected to exhibit zero mean, white noise characteristics, and statistical independence, which ensure the optimality of the Kalman filter. However, in the traditional uniform distribution model, the differential rotation of the Sun is neglected, changing the statistical properties of the innovations. To illustrate this effect, simulated innovations are presented under three scenarios: measurement error only, unmodeled system bias only, and the combination of both. Persistent biases or slow drifts in the innovations indicate inconsistencies between the measurement model and the true physical process, which can degrade filtering accuracy and compromise filter stability over long-term operations.

**Figure 4:**
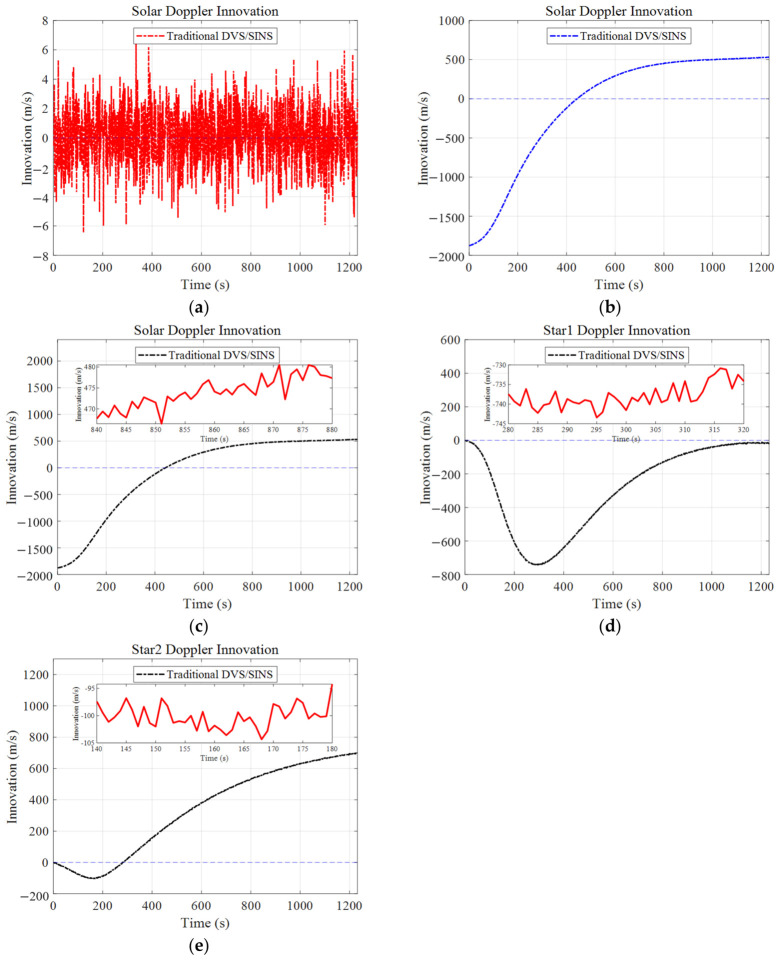
Innovations of the traditional DVS/SINS method under unmodeled system bias, measurement error, and their combination (**a**) Sun-measurement error; (**b**) Sun-system bias; (**c**) Sun-combined effect; (**d**) Star 1-measurement error; (**e**) Star 2-measurement error.

Figure 4 illustrates the temporal evolution of the innovations for the traditional DVS/SINS method under three scenarios. First, when only measurement error is present, the innovations of both the Sun and the two star channels exhibit zero mean random fluctuations without biases, which is consistent with the white noise characteristics required by the Kalman filter. Second, when only unmodeled system biases are introduced, the innovations in the Sun channel show significant non-zero static biases, indicating that these biases dominate the measurements. Furthermore, when both measurement error and unmodeled systematic biases are present, the Sun channel innovations still display the pronounced biases observed previously, suggesting that the impact of system biases on the innovation statistics far outweighs that of measurement error. In addition, due to the propagation of measurement model mismatches through the filter updates, the innovations of the two star channels in the traditional method also deviate from zero mean, demonstrating that unmodeled biases can contaminate multi-channel innovations and degrade overall filter performance.

**Figure 5:**
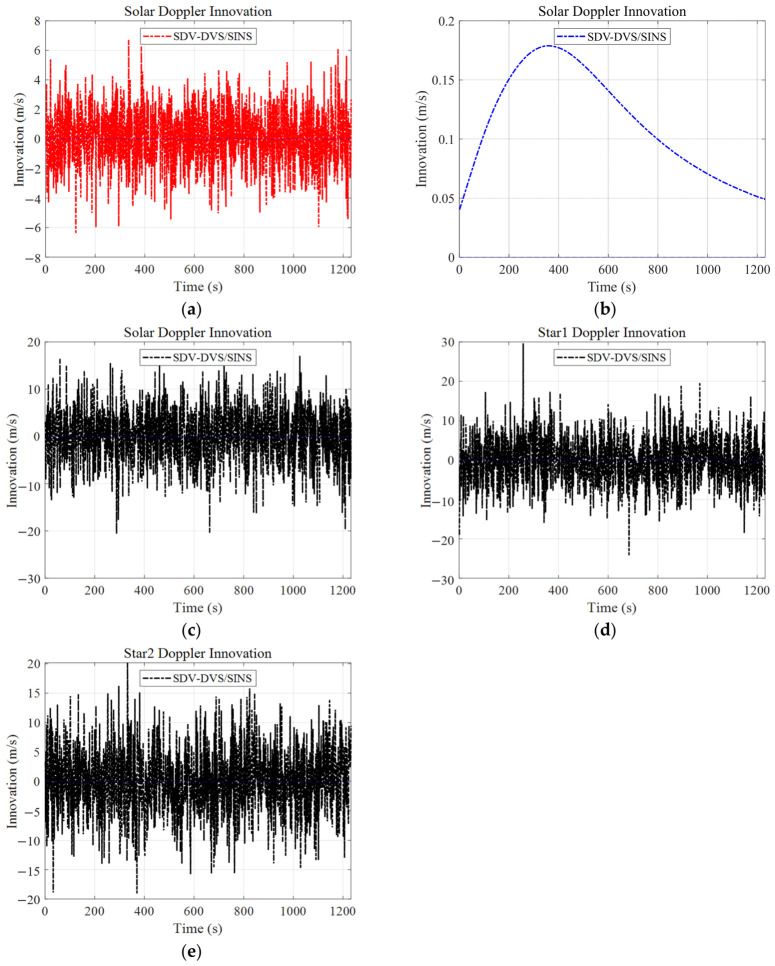
Innovations of the proposed SDV-DVS/SINS method under different errors (**a**) Sun: measurement error; (**b**) Sun: system bias; (**c**) Sun: combined effect; (**d**) Star 1: measurement error; (**e**) Star 2: measurement error.

Figure 5 and Table 4 show the innovations of the proposed SDV-DVS/SINS method under the same conditions. By modeling the true motion of the Sun’s disk observation points, the Sun channel innovations maintain zero mean random fluctuations in all three scenarios, with no system biases, confirming the consistency between the model and the measurements. Meanwhile, the innovations of the two star channels remain stable white noise, no longer affected by the propagation of Sun measurement errors. Overall, the proposed method significantly restores the statistical correctness of innovations, fundamentally eliminating the system biases caused by the uniform distribution assumption in the traditional method.

### 4.3. Navigation Performance Analysis

**Figure 6:**
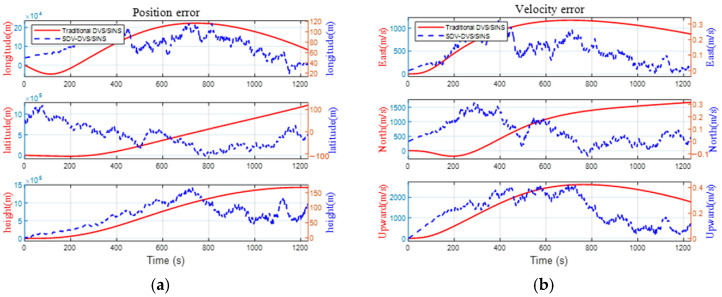

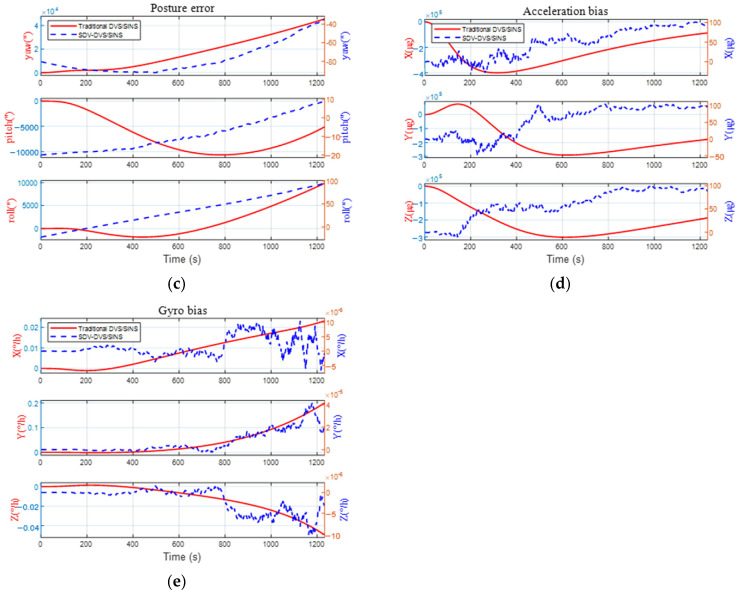
Navigation error of traditional DVS/SINS and the newly proposed method (**a**) position error; (**b**) velocity error; (**c**) posture error; (**d**) accelerometer bias; (**e**) gyroscope bias.

Figure 6 illustrates the simulation results comparing the traditional SINS/DVS integrated navigation method with the proposed solar disk velocity-based compensation for the unmodeled system bias method. The corresponding results are provided in Table 5. As shown in Figure 6a, affected by unmodeled system biases, the traditional method exhibits a pronounced and persistent growth in position errors, with longitude, latitude, and height errors rapidly expanding to the order of 10^4^–10^6^ m. In contrast, the proposed SDV-DVS/SINS method effectively maintains the position errors within a small bounded range throughout the simulation, with final errors remaining on the order of only tens to hundreds of meters. The velocity errors of the traditional method (Figure 6b) accumulate to several hundred to over a thousand meters per second, the proposed method keeps the east, north, and upward velocity errors below approximately 0.1 m/s, achieving more than three orders of magnitude improvement in velocity accuracy. This demonstrates that the proposed method can effectively utilize high-precision solar disk velocity information to compensate for unmodeled system biases. By maintaining accurate velocity estimates during the entire flight duration, the method fundamentally suppresses the quadratic accumulation of position errors. As seen from Figure 6d, the newly proposed method demonstrates a strong performance in estimating accelerometer bias. The traditional SINS/DVS method fails to converge to the true values due to the influence of unmodeled systematic biases. However, the DVS measurement does not contain posture information, the posture error and the gyroscope bias (as shown in Figure 6c,e) cannot be accurately corrected.

### 4.4. Impact Factor Analysis

#### 4.4.1. Measurement Error

**Figure 7:**
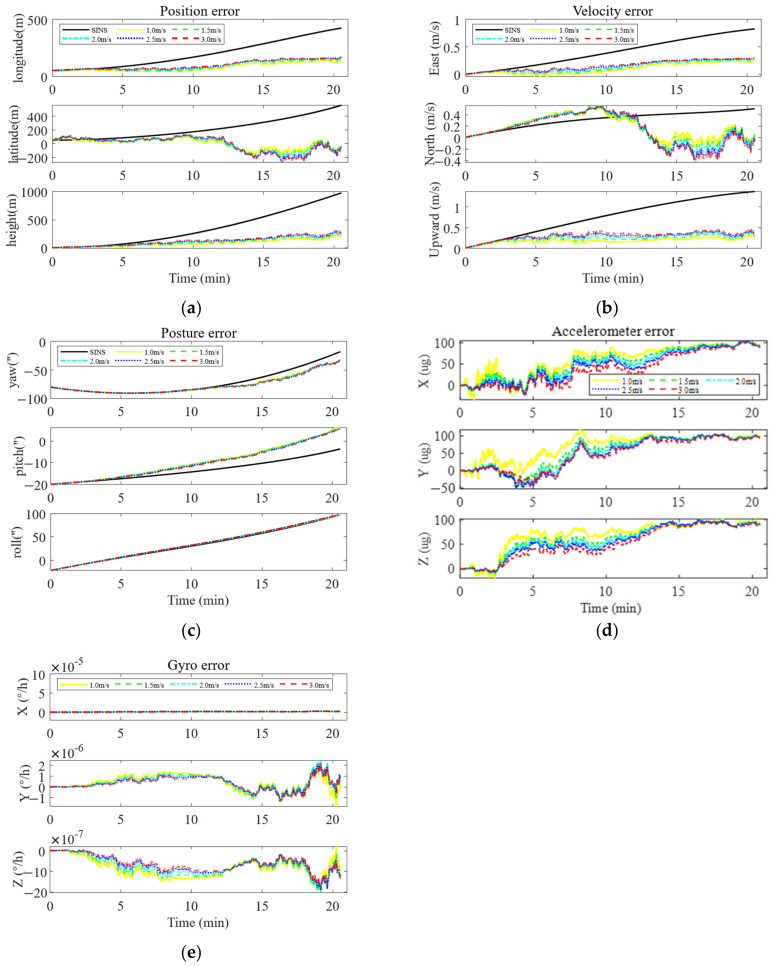
Navigation error of the newly proposed method with different measurement error (**a**) position error; (**b**) velocity error; (**c**) posture error; (**d**) accelerometer bias; (**e**) gyroscope bias.

Figure 7 shows the performance of the solar disk velocity-based compensation method under varying measurement errors, with the corresponding results presented in Table 6. It can be concluded from Figure 7 and Table 6 that as the measurement error increases, the error of the integrated navigation increases. This is because the measurement error decreases the accuracy of the measurement update in the UKF. With an increase in the measurement error, the state estimation error increases, which directly leads to an increase in the navigation error.

#### 4.4.2. Different Stars Observed in the Navigation

**Figure 8:**
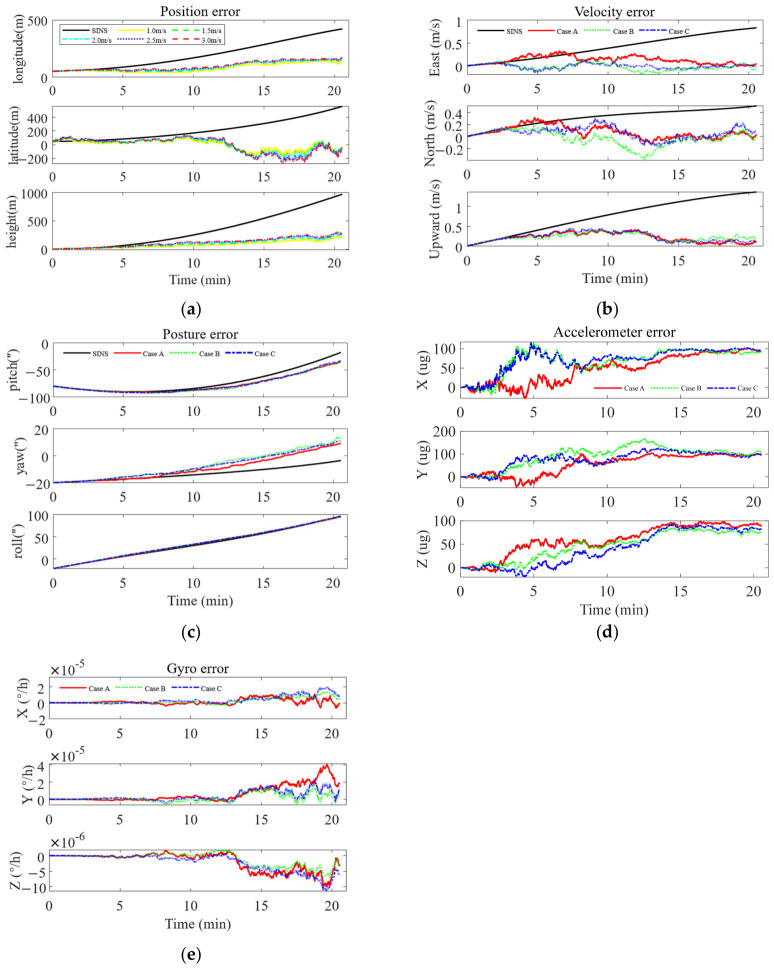
Navigation error of the newly proposed method with different stars (**a**) position error; (**b**) velocity error; (**c**) posture error; (**d**) accelerometer bias; (**e**) gyroscope bias.

This section analyzes the impact of different observed stars on navigation accuracy. As shown in Figure 8 and Table 7, the choice of stars has a significant effect on the navigation results. Specifically, since DVS measurements are scalar, selecting stars from different directions effectively provides velocity observations along corresponding directions, thereby enabling the correction of velocity errors. The better the geometric distribution of the stars, the stronger the system observability and the higher the navigation accuracy. These results highlight the importance of optimal star selection in the proposed method.

## 5. Discussion

This study shows solar differential rotation as a critical, yet frequently overlooked, source of systematic error in high-precision integrated navigation systems.

First, unlike traditional methods that assume a uniform radial velocity across the solar disk, our analysis demonstrates that the solar surface velocity is inherently non-uniform due to differential rotation. As derived in Section 2, this oversimplified velocity assumption introduces a deterministic velocity bias with a mean value of approximately 1.45 km/s and an upper bound of up to 2.07 km/s. Our innovation analysis (Figure 4 and Figure 5) confirms that this bias manifests as a persistent non-zero mean disturbance. This violates the zero mean and white noise assumptions of the Kalman filter, causing the filter to misinterpret model mismatch as actual state errors and leading to divergence. By accurately modeling the differential velocity across the observed solar disk, the proposed SDV method establishes a measurement model that accurately reflects the Sun’s rotational geometry. Consequently, this method eliminates the deterministic bias inherent in the traditional uniform-velocity models, removing the non-zero mean component from the filter innovations.

Second, by modeling the velocity of the observed solar disk point, the proposed method effectively compensates for unmodeled system bias. Simulation results in Section 4 demonstrate that, compared to the traditional method where errors diverge significantly, the proposed method successfully suppresses velocity errors to within the 10^−2^–10^−1^ m/s range. Furthermore, position errors are reduced from kilometer-level drift to the hundred-meter level.

However, several engineering challenges remain. Although the proposed method effectively eliminates the large-scale deterministic bias induced by solar rotation, the system’s performance in dynamic near-space environments warrants further investigation. Transient spectral disturbances such as those triggered by solar flares or coronal mass ejections can introduce anomalous frequency shifts. Future research will focus on incorporating adaptive neural network architectures to identify and mitigate these unpredictable spectral anomalies, thereby further enhancing the robustness of the SDV-DVS/SINS.

## 6. Conclusions

In this study, a high-precision multi-source integrated navigation framework via solar disk differential velocity is proposed to correct the unmodeled system bias (with a mean value of approximately 1.45 km/s and an upper bound of up to 2.07 km/s) introduced by assuming a uniform radial velocity across the solar disk in the traditional DVS/SINS integration navigation system. The newly proposed method constructs a velocity measurement model that accurately reflects the geometric structure of solar rotation by utilizing the Doppler velocity distribution across the solar disk, effectively compensating for the unmodeled system bias. In addition, measurement models for the DVS are established to enhance system observability and enable three-dimensional velocity error correction. As a result, the robustness and convergence of the filter are significantly improved, enabling rapid suppression of velocity errors and effectively mitigating the quadratic accumulation of position errors. To evaluate the effectiveness of the newly proposed method, simulation comparisons were performed between the traditional DVS/SINS and the newly proposed method. The results show that the newly proposed method significantly improves navigation accuracy. In comparison with the traditional DVS/SINS method, the longitude, latitude, and altitude errors are reduced from the kilometer scale to approximately a hundred-meter level. Likewise, the east, north, and upward velocity errors are suppressed to within the 10^−2^–10^−1^ m/s range. The newly proposed method effectively suppresses both velocity and position error growth in the navigation system and provides a feasible and effective solution for high-autonomy, dynamic flight navigation missions.

## Figures and Tables

**Table 1:** Parameters of the ideal trace.

Star Time	Horizontal Flight Path Angle	Azimuth	Velocity
23 April 2021 00:00.000	30 deg	90 deg	6 km/s

**Table 2:** Parameters of stars in this paper.

Star Number	Right Ascension (HMS)	Declination (DMS)	Radial Velocity (km/s)
Star1	14:15:52.5	$19^{\circ }9^{\prime }14.4^{\prime \prime }$	−5.0
Star2	9:27:50.2	$-8^{\circ }40^{\prime }39^{\prime \prime }$	−4.5
Star3	18:36:56.33	$38^{\circ }47^{\prime }1.17^{\prime \prime }$	−13.9
Star4	19:46:28	$10^{\circ }37^{\prime }24.3^{\prime \prime }$	−3.4
Star5	21:31:47.9	$-5^{\circ }33^{\prime }6.1^{\prime \prime }$	7.0
Star6	6:45:8.87	$-16^{\circ }42^{\prime }57.99^{\prime \prime }$	−7.6

**Table 3:** Parameters of the filter.

Parameter	Value
Process noise covariance	$Q={\left[q1,q1,q1,q2,q2,q2,0_{1\times 9}\right]}^{T}$
	$q1={\left(0.001/3600/180*pi\right)}^{2}$ $q2={\left(0.002\right)}^{2}$
Measurement noise covariance	$R=diag\left[r1,r1,r1\right]$
	r1=4
Trajectory duration	20.52 min
Filter period	1 s

**Table 4:** Mean Doppler measurement innovations (m/s).

Stars Used in the Navigation	Type of Disturbance	Traditional DVS/SINS	SDV-DVS/SINS
Sun	Measurement error	−0.04	0.03
	Unmodeled system bias	−742.3	0.11
	Combined effect	−738.6	−0.02
Star1	Measurement error	−0.01	0.02
	Unmodeled $\Delta v_{sys}$	−645.2	−0.03
Star2	Measurement error	0.05	−0.04
	Unmodeled $\Delta v_{sys}$	235.7	0.01

**Table 5:** Results of traditional DVS/SINS and SDV-DVS/SINS integrated navigation.

Methods	Position Error [m]			Velocity Error [m/s]			Posture Error [″]		
	Longitude	Latitude	Height	East	North	Upward	Yaw	Pitch	Roll
Traditional DVS/SINS	8.15 × 10^4^	1.21 × 10^6^	1.43 × 10^6^	872.74	1.66 × 10^3^	1.78 × 10^3^	4.55 × 10^4^	−5.10 × 10^3^	9.83 × 10^3^
SDV-DVS/SINS	37.12	−19.46	103.94	0.03	0.02	0.10	−34.78	8.91	94.93

**Table 6:** Result of SDV-DVS/SINS integrated navigation with different measurement error.

The Measurement Error	Position Error [m]			Velocity Error [m/s]			Posture Error [″]		
	Longitude	Latitude	Height	East	North	Upward	Yaw	Pitch	Roll
1.0 m/s	29.64	−10.54	75.36	0.01	−0.01	0.06	−35.46	9.02	94.71
1.5 m/s	34.01	−16.85	90.73	0.02	0.01	0.09	−34.96	8.99	94.79
2.0 m/s	37.12	−19.46	103.94	0.03	0.02	0.10	−34.78	8.91	94.93
2.5 m/s	40.49	−21.32	107.80	0.04	0.03	0.11	−34.66	8.82	94.92
3.0 m/s	44.98	−26.30	132.08	0.06	0.04	0.15	−34.33	8.75	95.25

**Table 7:** Result of SDV-DVS/SINS integrated navigation with different stars.

Case	Stars Used in the Navigation	Position Error [m]			Velocity Error [m/s]			Posture Error [″]		
		Longitude	Latitude	Height	East	North	Upward	Yaw	Pitch	Roll
case A	Sun + star1 + star2	37.12	−19.46	103.94	0.03	0.02	0.10	−34.78	8.91	94.93
case B	Sun + star1 + star3	−21.58	−34.31	125.58	−0.01	0.08	0.12	−33.94	10.95	95.18
case C	Sun + star2 + star3	−70.78	−96.36	176.74	−0.06	−0.02	0.19	−35.98	12.57	94.98

## Data Availability

The original contributions presented in this study are included in the article. Further inquiries can be directed to the corresponding author(s).
